# Modelling of Guillotine Cutting of a Cold-Rolled Steel Sheet

**DOI:** 10.3390/ma12182954

**Published:** 2019-09-12

**Authors:** Jarosław Kaczmarczyk

**Affiliations:** Institute of Theoretical and Applied Mechanics, Silesian University of Technology, 18A Konarskiego Street, 44-100 Gliwice, Poland; jaroslaw.kaczmarczyk@polsl.pl; Tel.: +48-32-237-2790

**Keywords:** steel sheet, finite element method, scanning electron microscopy, nonlinear analysis, fracture, ductile and brittle cracking, plastic and brittle zones

## Abstract

In this paper, the modelling of a cutting process of a cold-rolled steel sheet using a symmetrical cutting tool is presented. The fast-changing nonlinear dynamic cutting process was elaborated by means of the finite element method and the computer system LS-DYNA. Experimental investigations using scanning electron microscopy were performed and the results are presented in this work. The numerical results were compared with experimental ones. The comparison shows a good agreement between the results obtained by means of numerical modelling and those received from experimental investigations. The numerical simulations of the cutting process and the experimental investigations aimed to understand the mechanism of the cutting process. They serve as a highly professional tool for carrying out research investigating the behavior of complex nonlinear fast-changing dynamical cutting processes in the future.

## 1. Introduction

Nowadays, in the industry, highly effective and robust machines for the cutting of series of ultra-thin metal sheets that are usually arranged in bundles are ubiquitously used. Those professional machines are often equipped with cutting tools made of special hard materials, such as high-speed steel, cemented carbide, diamond, carbon nanotube, etc. However, the cutting tools, despite being made of super hard materials, are additionally coated with an ultra-thin overlay of extremely super hard materials. These mentioned materials are characterized by a hardness value exceeding 40 GPa according to the Vickers hardness scale. They are highly incompressible solids, with a higher electron density and high bond covalence. As a result of their unique properties, these materials are of great interest in many industrial areas, including but not limited to polishing abrasives, cutting tools, wear-resistant and protective coatings, etc. [1,2]. The cutting tools are often made of diamond, which is the hardest known material up to date, with a Vickers hardness value in the range of 70 to 150 GPa.

In industry, there are many professional machines used for the cutting of metal sheets arranged in bundles. Such cutting is highly efficient in comparison to cutting one separate metal sheet on cross cutters. At present, attempts have been made to cut as many metal sheets in the bundle as possible. However, the maximum allowable number of sheets in one single cut is limited, as well as the maximum height of the cut-off plates. Excess sheets arranged in a bundle might generate serious problems, with undesirable randomly happening defects on a cutting tool and in the cross-sections of cut-off sheets. Those defects are connected mainly with edge bending, vertical scratches, and burrs.

In this paper, the mechanism of cutting of one single plate with a symmetrical cutting tool was analyzed in order to study the behavior of metal cutting on the example of steel sheet C75S. This particular phenomenon belongs to dynamical fast-changing highly non-linear numerical simulations, which are quite difficult to model and analyze. It is not easy to explain the separation mechanism of sheets during the cutting process. In order to approach this problem, the physical model and the corresponding mathematical one were elaborated using a symmetrical cutting tool. It is rather impossible to observe all the details of the cutting process by means of the naked eye; therefore, this process is particularly fast changing. The objective of this work was focused on obtaining a better understanding of the mechanism of the cutting process. The experimental investigation using scanning electron microscopy was performed in order to verify the results of the numerical simulation of the cutting process. The numerical results were compared with experimental ones.

## 2. State of the Art

In case of designing buildings or mechanical structures, special attention should be paid to their safety regulations. The Huber–Mises reduced stress is a measure of security and should be lower than the permissible one [3]. If any physical phenomenon is repeated cyclically for a long time, then the fatigue life is important and should be taken into account due to the decreasing fatigue strength [4], especially when designing machines or mechanical devices.

The complete reverse (opposite) process is the modeling of the material separation. In this case, the process should be designed in such a way that the Huber–Mises reduced stress should exceed the allowable stress that appears in the direct cutting zone [5,6,7,8,9,10,11]. Otherwise, the material being removed would not be separated.

Nowadays, the works concerning the numerical modelling of machining processes using FEM (the finite element method) are very popular. Many literature positions describe numerical or experimental investigations of machining processes, such as turning [5], milling [6,7,8], boring [9,10], grinding [11], etc. Much less attention is given to covering mechanical cutting on guillotines [12,13,14,15,16,17,18].

During the modelling of a cutting process, the failure models [19,20,21,22,23], as well as fatigue testing [4], are quite important. The crucial issue is designing new industrial machines [24], as well as the selection of an appropriate finite element model [25,26,27,28,29,30]. The cutting of a single sheet [12,14] is much simpler in comparison with the cutting of a bundle of plates [13].

Elaboration of the physical failure models is rather difficult and that is why there are many different approaches. For example, Xiong and Xiao [31] extended the application range of the cohesive zone model, making it suitable for concrete fracture simulation both under tension and compression. Quantifying the effects of the different materials and interaction parameters studied in their paper contributes to revealing the relationship between a meso-scale fracture and microscopic behavior of concrete, which is rather quite similar to modelling fractures in metals [20,21,22,23].

A study of the cutting process by means of a non-symmetrical cutting tool was performed by Kaczmarczyk et al. [12,13]. In those papers, investigations concerning the cutting of one [12] and two separated sheets [13] were discussed in detail. The cutting tool was modelled as a non-symmetrical rigid body and the plate was cut as a deformable one. In the mentioned articles, it was proven that the undesired plastic zone embraces one third of the height of a sheet being cut, measured from the top of a sheet downwards. It means that the plastic zone corresponds to the area where many different defects, such as: edge bending, vertical scratches in the shape of craters, and burrs, happen simultaneously. However, the numerical cutting process is difficult to carry out because on the one hand, dynamical fast-changing phenomena occur [32,33,34,35,36,37,38] and on the other hand geometrical and material nonlinearities appear [26,27,28,29,30,39]. 

Many works address the issue of optimization in a broad sense using classical, evolutionary, or heuristic methods [40,41,42,43,44,45,46] in minimization. For example, Tiwari and Chakraborti [47] described a method of optimizing the layout of rectangular parts placed on a rectangular sheet to cut out various parts. They investigated two types of cutting problems. The first one was that guillotine cutting required metallic sheets, where each cut was made individually for one single sheet. The second one was that guillotine cutting was not essential, e.g., cuts that can be made using a punch, i.e., for such materials like paper or rubber. The optimization of the layout of rectangular parts was achieved by them with respect to two design objectives involving minimization of the length of the sheet required and the total number of cuts required to obtain all the parts from the mother sheet.

## 3. Methodology

In order to achieve the objective of this work relevant to understanding the mechanism of a cutting process, the following methodology was applied:

- Elaboration of a physical model of a cutting process;
- Adopting the mathematical model that corresponds to the mentioned physical model;
- Application of Columb and Moren’s friction model;
- Assumption of the material model and mechanical properties;
- Application of appropriate boundary conditions in loads and displacements;
- The finite element method and the computer system LS-DYNA (LSTC, Livermore, CA, USA) were used to model the cutting process;
- Experimental investigations based on observations of the microstructure of cross-sections of surfaces being cut using a scanning electron microscope with a cold field emission (FESEM) HITACHI S-4700 (Hitachi, Ltd., Tokyo, Japan) equipped with the energy dispersive X-ray (EDS) NORAN Vantage spectrometer (Noran Co, Vernon, CA, USA) were conducted;
- Comparison of gathered experimental data with the results of numerical simulations in order to verify the correctness of the assumed physical model was carried out.

### 3.1. Physical Model of the Sheet Being Cut

The questions concerning the numerical simulations and experimental investigations of the cutting process using a symmetrical and non-symmetrical cutting tool that should be answered are as follows:

(1) Is it possible to observe any geometrical and physical differences in the plate being cut that can be modelled as deformable during the cutting process?
(2) In case differences occur, what is the quantitative and qualitative nature of these differences?
(3) What does an equivalent Huber–Mises stress and strain distribution during the cutting process look like?
(4) How large are the plastic and brittle zones formed during cutting?
(5) Are the changes significant enough to prepare the optimization of the cutting process, taking into consideration the shape geometry of the blade of a cutting tool, in order to improve the quality of the surfaces of the plates being cut, which is understood as minimization of the size of the plastic zone, in the future?

To find the answers the formulated questions, the cutting process was elaborated using a symmetrical cutting tool. For realization of such goals, the following actions were performed:

(1) Preparation of the geometry of a symmetrical cutting tool;
(2) Elaboration of the geometry of a plate being cut (together with the remained pressure beam and worktable);
(3) Admission of the assumption of a physical material model;
(4) Assumption of rectilinear velocity for the cutting tool, resulting from the experimental investigation;
(5) Take into consideration the boundary conditions;
(6) Preparation of the physical model of a cutting process;
(7) Elaboration of the mathematical model based on the physical model;
(8) Execution of the numerical computations using the LS-DYNA computer system;
(9) Analysis and physical interpretations of the gained numerical results;
(10) Preparation of the experimental investigations using a scanning electron microscope;
(11) Analysis and physical interpretations of the gained experimental results;
(12) Comparison of the numerical and experimental results;
(13) Conclusions.

**Figure 1:**
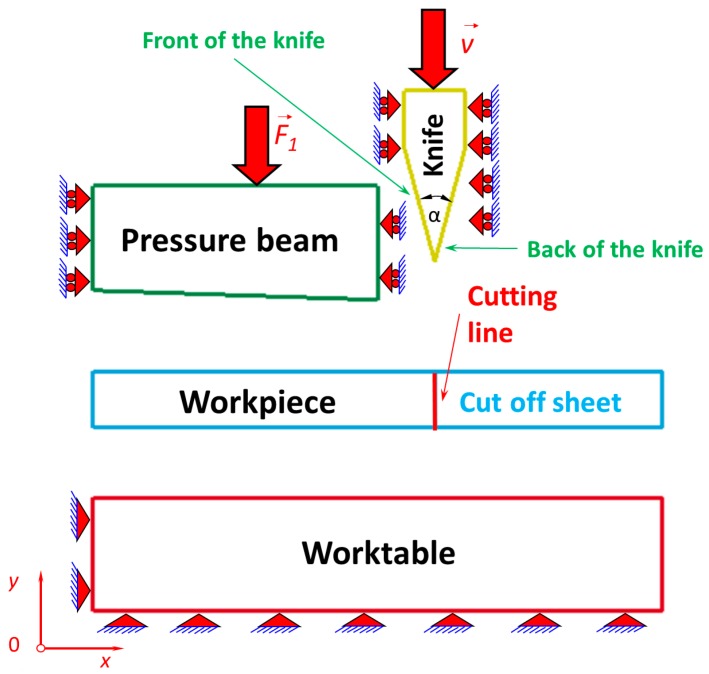
Physical model of the cutting process with a symmetrical cutting tool.

**Figure 2:**
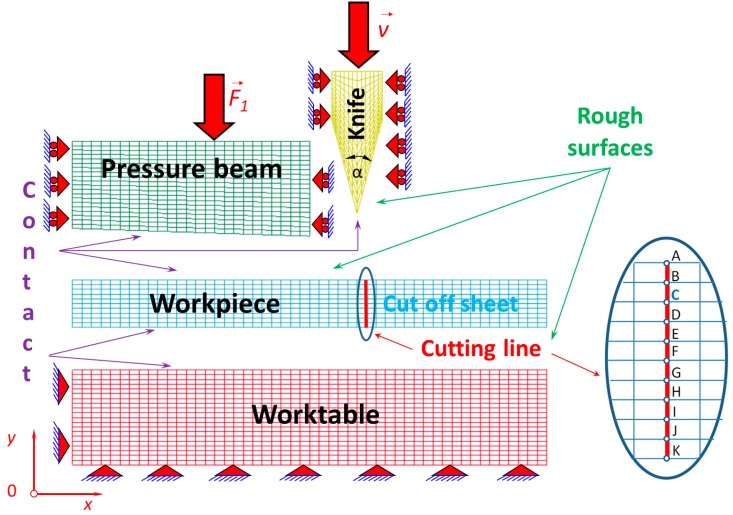
Discretization of the physical model into finite elements.

The physical model of the cutting process with a symmetrical cutting tool is shown in Figure 1 and discretization of the physical model into finite elements is shown in Figure 2. The following dimensions of the cutting tool were assumed: 30 × 25 × 6 mm (height × width × thickness) with an apex angle of 30°. The dimensions of the sheet being cut were as follows: 0.1 × 12.5 × 1 mm (height × width × length). The pressure beam size was 22 × 60 × 200 mm (height × width × length) with an inclination of 1:30. In this work, the edge of the blade of the cutting tool was treated as sharp, which corresponds to a brand new one or a blade after grinding. The cutting tool and pressure beam were scaled down to the dimensions shown in Figure 1 and Figure 2, except the sheet being cut. The apex angle of the cutting tool and the inclination of the pressure beam were conserved. This aimed to make the visualization of the process more legible and had no influence on the numerical results. The maximum constant velocity of the cutting tool was *ϑ* = 10 mm/s. The size of the mesh of the sheet being cut was assigned 0.02 mm horizontally and 0.01 mm vertically, respectively. The cutting tool was elaborated as a rigid body because it was made of high-speed steel (NC 10/1.2201) and its dimensions were substantially large compared with the ultra-thin plate being cut (thickness 0.1 mm) made of cold-rolled steel C75S. The pressure beam and the worktable were very stiff too, compared with the earlier mentioned plate being cut, which why they were also elaborated as rigid bodies. The plate being cut and its cutting process play a highly important role and that is the reason why it was modelled as deformable. The elaborated cutting process is highly nonlinear, not only because of the geometrical nonlinearities (contact) but also because of the material nonlinearities of the plate being cut. The unilateral constraints, generally well known for contact, were imposed on all nodes belonging to the surfaces that were in mutual contact between bodies during the numerical simulations of the cutting process. The unilateral constraints are imposed between the following parts (Figure 1 and Figure 2):

- The blade of a cutting tool and the plate being cut;
- The blade of a cutting tool and the worktable;
- The pressure beam and the plate being cut;
- The plate being cut and the worktable.

In order to allow the progress of the cutting process, the failure physical model was required. In the current work, it consisted in separation of the nodes belonging to the cutting line. If the equivalent Huber–Mises strain in the sheet being cut exceeded the strain (*ε_f_* = 0.15) corresponding to the failure during the unilateral tensile test, the separation was possible. In the opposite case, when the equivalent Huber–Mises strain was less than the failure strain (*ε_f_*), the nodes lying on the cutting line were not submitted to any separation. The assumed failure model is important, therefore it allowed the cutting tool to penetrate through the material being cut and in consequence to cut a piece of metal into two separate parts.

The proposed element size was chosen on the basis of the experiment as a compromise between the number of elements and the time consumption required for complex numerical calculations.

In practice, solution difficulties usually arise only when the finite element discretization is very fine, and for this reason, the matter of infinite stresses under a concentrated load is frequently ignored. Much finer discretization leads to stress singularities at the point where the tip of a blade touches the sheet being cut. Additional errors might be caused also by the computer approximation of very small floating-point digits because the numbers are processed by rounding. However, in general, the finer the mesh, the better the results (small errors), but the time of calculations increases. Therefore, the balance between the accuracy and the time of computations should be established. If the size of the elements in the model is too large, the time of computations decreases, but the value of errors increases. It is usually caused by the assumed linear shape function and the number of nodes. In the current work, the proper size of the mesh was established on the basis of the experiment and the numerical calculations were verified with the experimental data.

**Figure 3:**
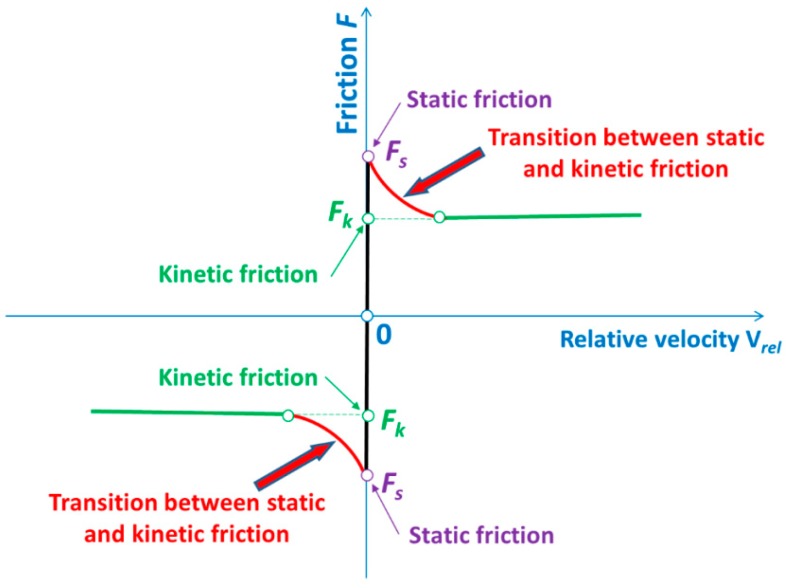
Friction force versus relative velocity.

Between all mentioned contact surfaces, the Coulomb and Moren’s model of friction was taken into consideration [48]. Friction is a physical phenomenon that is widespread in nature and has a major influence on the character of mechanical systems’ work. Coulomb and Moren’s friction model is most commonly used for friction modelling in mechanical systems, and was employed in this work (Figure 3). The static and kinetic coefficient of friction was assumed for steel (for grinded and dry surfaces) according to the literature data [48]. The static coefficient of friction equals (*µ_s_* = 0.22) and the kinetic coefficient of friction was established (*µ_d_* = 0.11), respectively, for all touching surfaces.

The effect of speed on the friction force, as well as the transition between the static and kinetic friction, were taken into consideration. The exponential decay coefficient, *φ* = 500, was assumed [49]. The relationship between the friction force (*F*) and coefficient of friction (*µ*) according to Coulomb and Moren’s law is expressed by the following well known Equation (1) [48,49]:

(1) F=μ·N,

where: *µ*—coefficient of friction, *N*—normal force.

The normal force is perpendicular to the surface, e.g., for a sheet being cut and the worktable. It was counted in each iteration by means of the computer system LS-DYNA. The coefficient of friction (*µ*) is expressed by the following Equation (2) [49]:

(2) $$\mu =\mu_{d}+\left(\mu_{s}-\mu_{d}\right)\cdot e^{-\varphi \cdot |V_{rel}|},$$

where: *µ_s_*—static coefficient of friction, *µ_d_*—dynamic coefficient of friction, *φ*—decay coefficient, *V_rel_*—relative velocity.

The boundary conditions were imposed on a worktable (Figure 1 and Figure 2) in such a way that the worktable was stationary. It means that motion along the *x* and *y* axes, respectively, was blocked. In nodes belonging to the bottom and to the vertical wall on the left-hand side of the worktable, two degrees of freedom were taken away in a node (along the *x* and *y* axes, respectively). Such an applied support allowed neither translational nor rotational motion of the whole worktable, which was thus treated as a rigid body. This means that the worktable was stationary, distinct from the deformable plate being cut off, on which no boundary conditions were imposed except the unilateral constraints that may have blocked the motion of the plate being cut. The exerted forces acted on the plate being cut by a cutting tool moving vertically as the first rigid body, the pressure beam acting downwards as the second rigid body, and finally the worktable being stationary as the third rigid body by friction and normal forces (reactions). So, the plate being cut thus remained stationary as long as the cutting tool moved through the height of the metal sheet. Then, the workpiece (the plate being cut) could be divided into two pieces according to the failure model presented earlier.

### 3.2. Material

**Figure 4:**
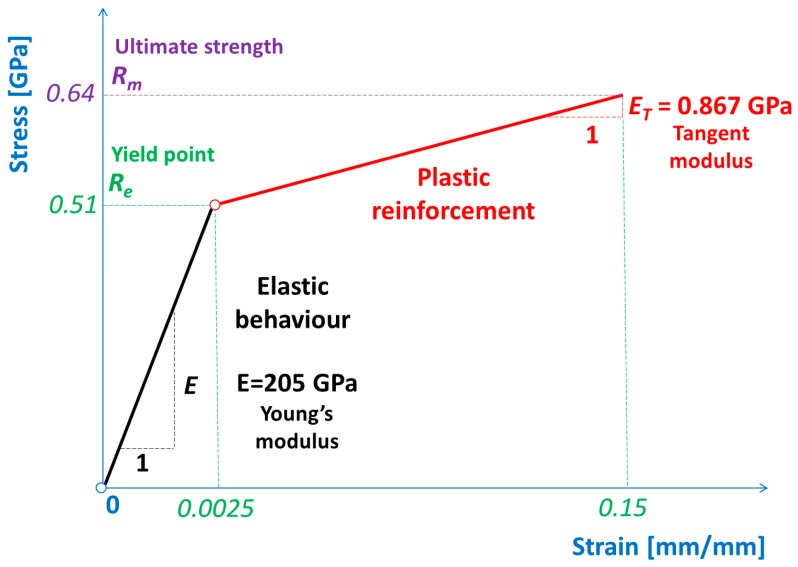
Physical bilinear elastic–plastic material model.

The bilinear elastic–plastic material model of a sheet made of C75S (high-strength steel) was adopted for performing the numerical simulations and is presented in Figure 4. The details of the material properties for which the numerical calculations were performed are juxtaposed in Table 1 [2,12,13]. The assumed bilinear material model belongs to the simplest possible ones describing the nonlinear stress–strain behavior [39]. It consists of two linear stress–strain characteristics, which are connected with each other. The first one starts at the origin of the Cartesian coordinate system and describes the tangent of the inclined angle (*α*) with respect to the abscissa. It expresses the elastic modulus, also called the Young’s modulus (*E*), and is usually represented by Equation (3):

(3) E=tan(α).

The second one starts at the yield point (*R_e_*) and describes the tangent of the inclined angle (*β*) with respect to the abscissa. It expresses the tangent modulus (*E_T_*), which usually characterizes the plastic reinforcement of material and is often represented by Equation (4):

(4) $$E_{T}=\tan\left(\beta \right).$$

## 4. Results and Discussion

This chapter includes the results of the numerical simulations and experimental investigations in the form of scanning electron images, as well as the analysis and comparison of the numerical and experimental results that validates the obtained findings.

### 4.1. Numerical Simulations

The cutting process using a symmetrical cutting tool was modelled by means of the finite element method and computer system LS-DYNA [49].

**Figure 5:**
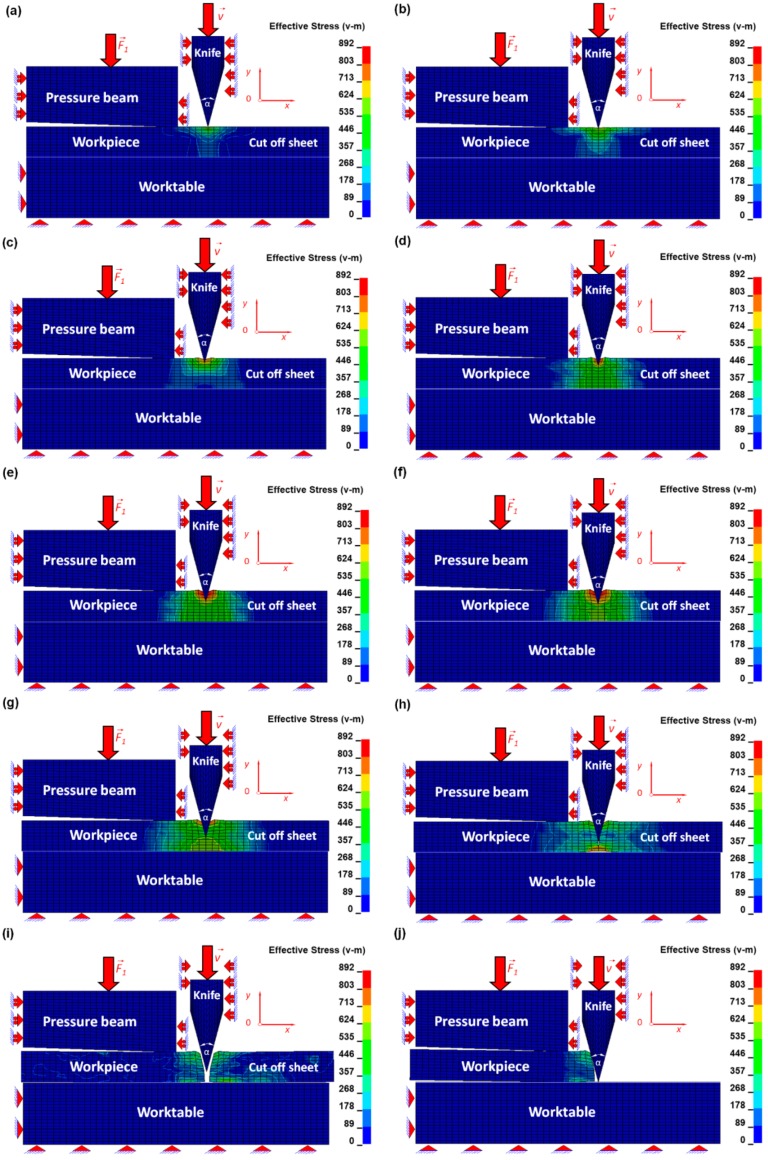
Distribution of the equivalent Huber–Mises stresses [MPa] for arbitrarily selected time instances (ms): (**a**) *t* = 2.04, (**b**) *t* = 2.4, (**c**) *t* = 3.4, (**d**) *t* = 4.4, (**e**) *t* = 5.4, (**f**) *t* = 6.4, (**g**) *t* = 7.4, (**h**) *t* = 8.4, (**i**) *t* = 8.42, (**j**) *t* = 12.

**Figure 6:**
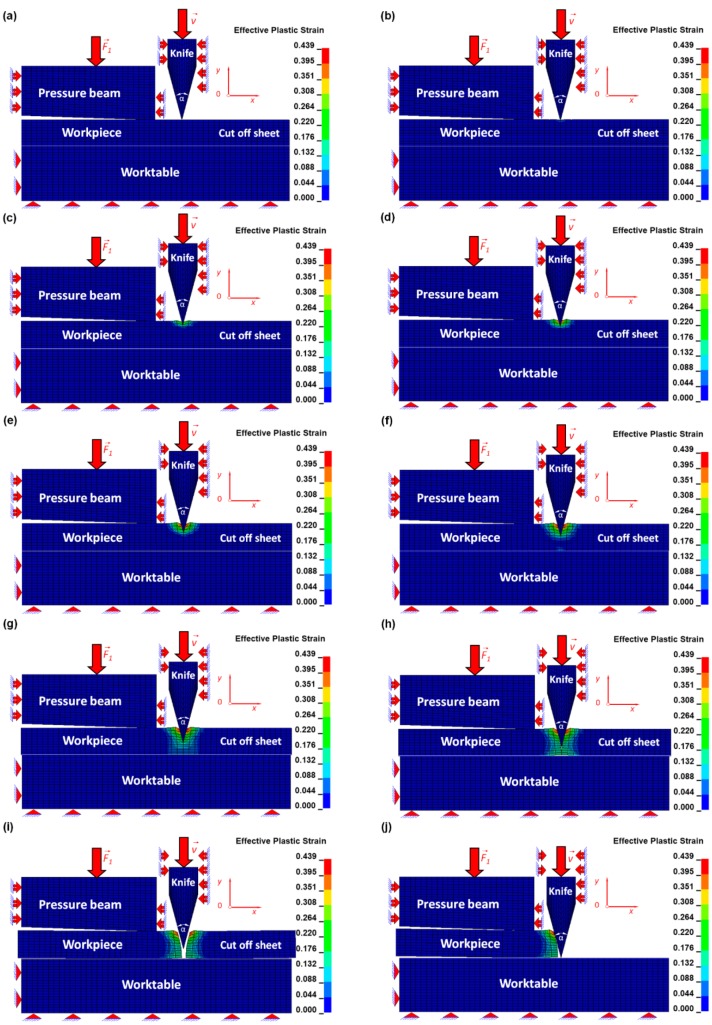
Distribution of the equivalent Huber–Mises plastic strains for arbitrary selected time instances (ms): (**a**) *t* = 2.04, (**b**) *t* = 2.4, (**c**) *t* = 3.4, (**d**) *t* = 4.4, (**e**) *t* = 5.4, (**f**) *t* = 6.4, (**g**) *t* = 7.4, (**h**) *t* = 8.4, (**i**) *t* = 8.42, (**j**) *t* = 12.

The numerical results were juxtaposed for several successive time intervals in order to facilitate the investigation of the mechanism of the cutting process using a symmetrical cutting tool (Figure 5 and Figure 6).

The cutting process was divided into two steps that were carried out according to the velocity profile:

- In the first step, the pressure beam compresses the sheet;
- In the second step, the cutting tool moves in order to cut off the sheet.

The pressure beam is located a few millimeters over the ultra-thin sheet and starts moving from its original position vertically downwards to the sheet until they meet. The motion begins at a certain initial time (*t* = 0 ms) and covers three successive stages:

- The first one is connected with a gradual smooth starting-up motion from the original position downwards until the pressure beam attains a maximum rectilinear velocity;
- The second one concerns the rectilinear motion, with a maximum constant velocity (12 mm/s) until it reaches certain a priori defined conditions;
- The third is tied with a deceleration of the pressure beam, which moves towards the ultra-thin plate being cut.

The rectilinear motion of the pressure beam slows down according to the gradual smooth characteristics until it reaches a zero velocity and develops a small compressing force exerted on the plate being cut. It is worth mentioning that the developed force is so small that it might be neglected during the cutting process. Nevertheless, it does not mean that the cutting process can be executed without the pressure beam. It only suggests the exerted force might be treated as insignificantly small and finally, that it can be assumed as being equal to zero. Moreover, the pressure beam plays a substantial role and is extremely important because it does not allow the sheet being cut to move during cutting and it also prevents the plate from bending. Furthermore, the pressure beam allows for cutting, with a certain gap located between the pressure beam and the blade of the cutting tool. The earlier investigation presented in the authors’ article [16], which was focused on non-symmetrical cutting, clearly demonstrates that the smaller the gap is, the smaller the force needed to separate the plate being cut. Additionally, the experimental investigation confirms that the smaller the gap is, the higher the roughness observed.

When the pressure beam stops, the cutting tool located over the ultra-thin steel sheet starts moving downwards in order to cut off the sheet. The motion begins from time (*t* = 2 ms). The cutting tool starts moving gradually from its original position downwards until the cutting tool attains maximum rectilinear velocity and then it cuts a sheet with a constant velocity (10 mm/s). After the sheet is cut off, it slows down and stops on the surface of the worktable and then returns to its original position. Then, the process is repeated.

During cutting, two characteristic zones are formed. The first one is connected with the plastic zone and the second with the brittle one. It is important to mention that the plastic zone denotes cutting with undesired effects, like edge bending, easy creation of burrs, and scratches in the shape of vertical craters. So, the plastic zone has many disadvantages of the mentioned features. It is obvious that such an undesirable zone should possibly be eliminated or, if it is impossible, it should be subjected to minimization using the classical optimization [41,42], evolutionary [43,44], heuristic, or hybrid method. The second mentioned zone is the brittle one. This zone is highly desirable, therefore it is accompanied by no burrs, vertical scratches in the shape of vertical craters, and edge bending. This particular zone is caused by progressive cracking during the cutting process and it is very important to pay particular attention to this brittle zone.

If it is possible to find the direct cause responsible for the generation of this kind of desirable zone and enlarge it, then in such a case, it could be plausible to control the size of this advantageous zone. A larger zone means better-quality cut surfaces of the sheet being cut, which is understood as the minimum possible roughness, minimum bending edges, no vertical scratches, and so on. The question is “if it is possible, by changing the shape of a cutting tool from non-symmetrical to symmetrical, to influence the size of the plastic and brittle zone in such a way that the brittle zone (highly advantageous one) is enlarged while simultaneously reducing the plastic one (highly disadvantageous and responsible for many kinds of defects during cutting)?”.

The conducted numerical simulations and experimental investigations ensure that it is possible to increase the size of the brittle zone.

It is important to mention that a fracture is the separation of an object or material into two or more pieces under the action of stress. The fracture of a solid usually occurs due to the development of certain displaced discontinuity surfaces within the solid. If a displacement develops perpendicularly to the surface, it is called a normal tensile crack or simply a crack; if a displacement develops tangentially to the surface, it is called a shear crack, slip band, or dislocation. In this paper, only fracture in the normal direction was considered.

Brittle fractures occur with no apparent deformation before the event; ductile fractures occur when visible deformation occurs before separation. Fracture strength or breaking strength is the stress when a specimen fails or fractures. A detailed understanding of how fracture occurs in materials may be assisted by the study of fracture mechanics.

The most interesting research is not only focusing on the beginning and ending of the cutting process but also on investigation of the all remaining stages. The beginning of the cutting process is tied with Hooke’s law because the stresses and the corresponding strains are less than the yield point (Figure 4), which is presented in Figure 5a,b and Figure 6a,b. Therefore, the symmetrical cutting tool begins touching the ultra-thin sheet.

In this current article, the reduction of the plastic zone, as well as the growth of the brittle one, which is influenced by the change of the geometry of the cutting tool from non-symmetrical (discussed in detail in the previous co-authors’ articles [12,13]) to symmetrical, was presented. In Figure 5c,d and Figure 6c,d, the beginning of the formation of the plastic zone is shown.

The progressive equivalent Huber–Misses stresses and strains develop and exceed the yield point, so the plastic zone starts forming. During this particular state, almost all undesirable defects intensify and are connected with edge bending, roughness, and vertical scratches. Finally, the plastic zone ceases to exist, which is presented in Figure 5e,f and Figure 6e,f. However, in Figure 5g and Figure 6g, the beginning of a new stage called the brittle zone starts developing. It is tied with the beginning of the brittle fracture. During this particular stage, the edge bending, burrs, and vertical scratches cease to dominate. The cut surfaces are clear without any defects and even if they happen, they occur rather sporadically, usually with a substantially small probability. This particular cutting zone is still developing and can be observed in Figure 5g,h and Figure 6g,h. In a brittle fracture, no apparent plastic deformation takes place before the fracture. Brittle fracture typically involves little energy absorption and occurs at high speeds—up to 2133.6 m/s in steel. In most cases, brittle fracture will continue even when loading is discontinued.

In Figure 5i,j and Figure 6i,j, the sheet is shown as being separated into two pieces. The first one on the left is located between the pressure beam and the worktable, and behaves as deformable and stationary. However, the second one, located on the right of the cutting line, begins moving like a rigid body despite the fact that it is treated as a deformable body. This behavior of the piece of the plate being cut is exerted by the cutting tool, which cuts the plate and lets it travel freely as a consequence. During this stage of the process, only the inertial force acts on the plate. The mass of the cut-off sheet is small due to the small dimensions of 0.4 × 0.1 × 12.5 mm (length × thickness × width) of the plate so the change in the equivalent Huber–Mises stress and plastic strain is small and that is why it is possible to treat this plate as an approximately rigid one, where there is constant stress and strain caused by plastic deformation and produced by the cutting tool.

It seems that the size of the plastic and brittle fractures depends on the geometry of the cutting tool. A comparison of the results of the previous co-authors’ works concerning the cutting process using a non-symmetrical cutting tool [12,13] and the current study (the manuscript) implies that the size of the plastic and brittle zones changes relative to the shape of the cutting tool. The cutting process using the symmetrical cutting tool results in a reduction of the plastic zone in the material being cut. In the future, the author plans further investigation concerning the optimization process, allowing for control of the size of the discussed zones and focusing on further reduction of the plastic zone using a different geometry of the cutting tool, for example, various values of the apex angle of the cutting tool.

The numerical calculation for the sharp blade of the cutting tool and for an arbitrarily assumed small radius on the tip of the cutting tool equal to 1 µm was performed. The corresponding maximum equivalent Huber–Mises stress values in the sheet being cut were: 891.75 and 860.77 MPa, for the sharp and round blade, respectively. The stress change reached up to 3.47% for the round blade with respect to the sharp blade.

### 4.2. Microscopic Observations

Experimental investigations were carried out using a scanning electron microscope with the cold field emission (FESEM) HITACHI S-4700 equipped with an energy dispersive X-ray (EDS) NORAN Vantage spectrometer.

**Figure 7:**
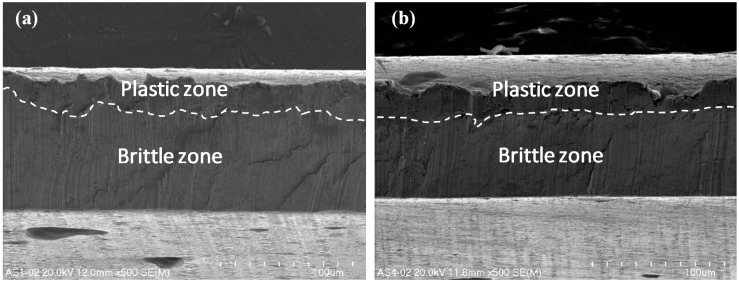
The surfaces of the steel sheet being cut under magnification x500 from the front (**a**) and from the back of the cutting tool (**b**).

**Figure 8:**
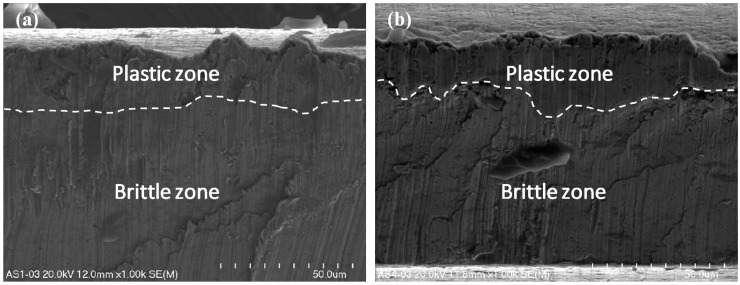
The surfaces of the steel sheet being cut under magnification x1000 from the front (**a**) and from the back of the cutting tool (**b**).

**Figure 9:**
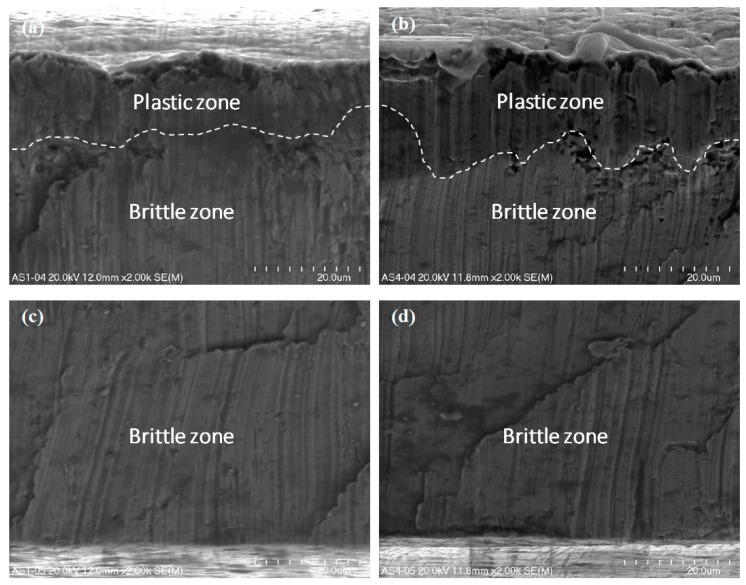
The surfaces of a steel sheet being cut under magnification x2000 from the front (**a**,**c)** and from the back of the cutting tool (**b**,**d**).

The ultra-thin metal sheet cutting process led to the occurrence of two types of fracture in the material. There is a crucial difference in the nature of the fracture in terms of the front side and the back side of the cutting tool (Figure 1). Detailed observation of the surface of the ultra-thin metal sheet after cutting was conducted under magnification (x500, x1000, and x2000) and the presence of the ductile and brittle zones was detected (Figure 7, Figure 8 and Figure 9). The plastic zone is always located in the upper part of the observed cross-section, whereas the brittle zone appears in the lower part. The border between those two zones is located at circa 1/5 of the sheet height measured from the top downwards independently of the place of the cross-section of a sheet being cut.

Micro-voids of various sizes are scattered all over the plastic zone. They are two different shapes, either spherical or ellipsoidal. They form due to the presence of nucleating sites and therefore they are located close to one another. They frequently coalesce together before growing to a larger size [50]. In the lower part of the sheet being cut below circa 1/5 of its height measured from the top downwards, numerous brittle walls were observed, but no micro-voids in the shape of dimples were detected.

The nature of the fracture in the cut surfaces changes depending on the location of the cutting tool. If the process is observed from the front of the cutting tool, the cross-sections are homogenous (Figure 7a, Figure 8a and Figure 9a,c).

In the opposite case, when the observation was conducted from the back of the cutting tool, the cross-sections are non-homogenous (Figure 7b, Figure 8b and Figure 9b,d). Typical features of a brittle fracture were observed in the shape of vertical scratches, shear planes, and some cracks. The separation of metal sheets resulting from decohesion occurred without plastic deformations.

In this current work, only one sheet was taken into consideration using a symmetrical cutting tool. The microstructure of the cross-sections is similar to that presented in a previous work [12] concerning the cutting of single sheet with a non-symmetrical cutting tool. The main difference consists in the size of the plastic and brittle fracture. In this particular case, when using a symmetrical cutting tool, the size of the plastic zone is significantly smaller in comparison with the plastic region produced by cutting using a non-symmetrical cutting tool.

**Figure 10:**
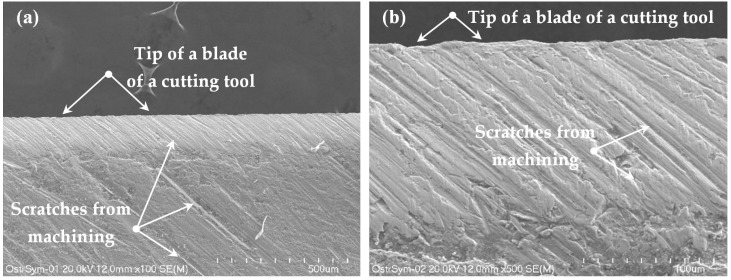
The surface of a blade under magnification: (**a**) x100, (**b**) x500.

The tip of a blade of a cutting tool is shown in Figure 10. It is common knowledge that the tip of a blade has the shape of a straight line, as presented in Figure 10a, under small magnification (x100). Nevertheless, the higher the magnification is (according to Figure 10b), the more indented the curve seems to be.

The mentioned curvilinear shape of the tip of the blade causes vertical scratches, which are produced during cutting and are presented in Figure 7, Figure 8 and Figure 9. However, on the cut surfaces, there are also scratches formed at an angle of circa 45°. The suggested question is where are they from?

The cutting tool moves only downwards along the vertical direction, but some scratches occur at a slant. To answer this question, observation of the external surface of a cutting tool was required (Figure 10). On its external surface, the inclined scratches are visible. They result from machining and they are partially reflected in the cross-section of the sheet being cut.

### 4.3. Comparison of the Numerical and Experimental Results

**Figure 11:**
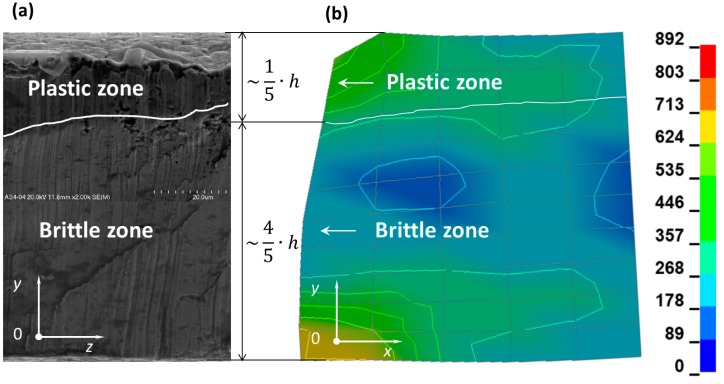
Comparison of the experimental results (**a**) with numerical ones (**b**).

**Figure 12:**
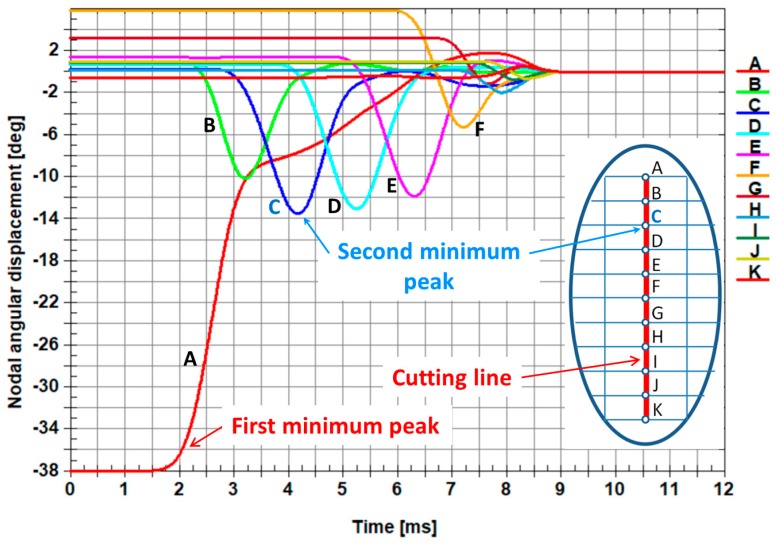
Comparison of the change of the angular displacement versus time in nodes belonging to the cutting line. The letters from A to K represent the nodes belonging to the cutting line (Figure 1 and Figure 2).

**Figure 13:**
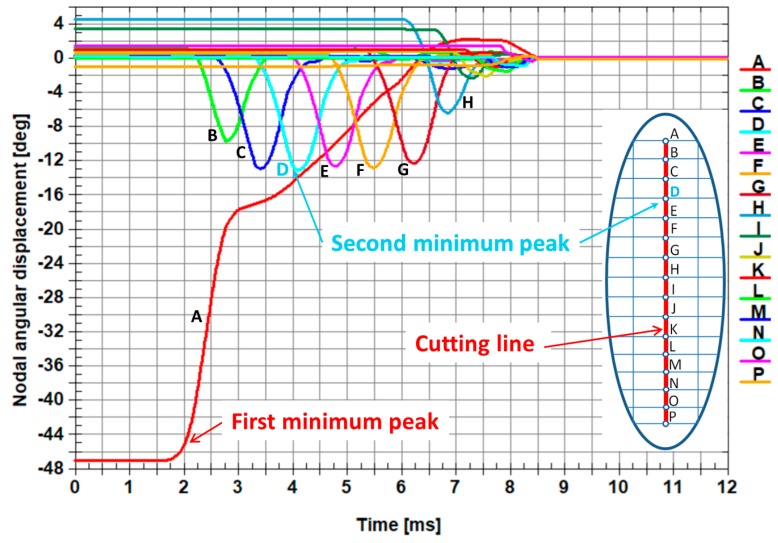
Comparison of the change of the angular displacement versus time in nodes belonging to the cutting line. The letters from A to P represent the nodes belonging to the cutting line for the sheet being cut divided into the doubled number of nodes with respect to the model shown in Figure 2.

The comparison of the numerical results and experimental ones is presented in Figure 11. The numerical results are shown in the *xy* plane (Figure 11b), but the experimental ones present the cross-section of sheet being cut, which is represented by the normal parallel to the *x* axis (Figure 11a). It provides evidence that the border (marked by a white curve) located between the plastic fracture and the brittle crack exists and can be easily determined. This is also confirmed by the change of the angular displacement versus time presented in Figure 12 and Figure 13.

The first minimum peak concerns the angular displacement of the first node from the top on the cutting line (node A). It is worth noting that the second minimum peak of the angular displacements corresponds to the transition from the plastic zone to the brittle one (node C in Figure 12 and node D in Figure 13). It is also visible in Figure 11, where a white curve divides the surface of the sheet being cut into two characteristic zones.

The plastic zone occupies circa 1/5 of the height of the sheet being cut measured from the top downwards and the brittle zone encompasses circa 4/5 of the height of the sheet being cut measured from the bottom upwards.

In order to prove that the current elaborated physical model consists of the optimal number of elements, the next one (advanced), with a doubled number of nodes of the sheet being cut, was created. The size of a single finite element was reduced from 0.02 mm horizontally and 0.01 mm vertically for the current model to 0.01334 mm horizontally and 0.00667 mm vertically for the advanced one, respectively. The numerical calculations for the advanced model were very time consuming (about four times longer) in comparison with the current model. The results of the numerical calculations are presented in Figure 12 for the current model and in Figure 13 for the advanced one. Both models provide the same information about localization of the border between the plastic and brittle zone, which corresponds to the second global minimum peak of the angular displacement. The results from both models show that the border between the plastic and brittle zone is located at circa 1/5 of the height of the sheet being cut measured from its top downwards. It proves that the size of the single finite element is optimal for the current model; therefore, the comparison of the results between the current model and the advanced one gives almost the same results.

The microscopic observation and the numerical results confirm there is very good agreement concerning the size, as well as the location, of the plastic zone, brittle area, and the border between them.

## 5. Conclusions

This paper investigated the mechanism of the cutting process by using a symmetrical cutting tool, which constitutes an extremely difficult problem because it is based on dynamical fast-changing phenomena that are impossible to observe using the naked eye. Numerical simulations were performed using the finite element method and the computer system LS-DYNA. The explicit analysis was carried out taking into account the material and geometrical nonlinearities. The experimental investigation was also conducted by means of scanning electron microscopy in order to study the cross-section surfaces of the ultra-thin metal sheet being cut. Finally, a comparison of the numerical and experimental results demonstrated quite good agreement. On the basis of the thorough analysis, the following conclusions were drawn:

- The stresses and strains are symmetrically distributed with respect to the vertical line passing through the tip of the blade of the cutting tool;
- The equivalent Huber–Mises stress and strain distributions during cutting are different at the beginning and at the end of the cutting process. At the beginning of the cutting process, the stress and strain are concentrated in the direct cutting zone, which comes from shearing and results in ductile cracking. At the end of the cutting process, the shearing ceases to act and the tension starts to dominate, resulting in the occurrence of a brittle fracture, the mechanism of which is similar to the cracking during the uniaxial tensile test;
- The surfaces of the sheet being cut are divided into two characteristic zones:
  ○ The first one (undesired) is connected with the plastic fracture accompanied by many defects, such as edge bending, scratches in the shape of vertical craters, burrs, etc.;
  ○ The second one (highly desired) concerns brittle fracture with no defects. The surfaces are characterized by high smoothness and low roughness, without vertical scratches or burrs;
- The comparison concerning the numerical results and experimental ones provides evidence that the border between the plastic fracture and brittle crack exists and can be determined;
- The plastic and brittle fracture depends on the cutting process and on the material being cut too, however, these two effects were taken into consideration simultaneously in the numerical simulations and experimental investigations. Within the limited range of velocities of the cutting tool, varying up to 10 mm/s, the following two conclusions might be drawn:
  ○ The plastic zone occupies circa 1/5 of the height of the sheet being cut measured from the top downwards and hence, edge bending, vertical scratches, and burrs occur in this particular area;
  ○ The brittle zone encompasses circa 4/5 of the height of the sheet being cut measured from the bottom upwards and that is why there are no defects, such as edge bending, vertical scratches, and burrs, in this specific region.

## Figures and Tables

**Table 1:** Material properties for the ultra-thin metal sheet being cut (steel C75S).

No	Name of the Material Properties	Symbol	Value [2,12,13]
1	Young’s modulus	*E*	205 GPa
2	Poison’s ratio	*ν*	0.28
3	Kirchhoff’s modulus	*G*	80 GPa
4	Tangent modulus	*E_T_*	0.867 GPa
5	Failure strain	*ɛ_f_*	0.15
6	Yield stress	*R_e_*	0.51 GPa
7	Ultimate tensile strength	*R_m_*	0.64 GPa
